# High-definition transcranial direct current stimulation (HD-tDCS) in major depressive disorder with anxious distress—a study protocol for a double-blinded randomized sham-controlled trial

**DOI:** 10.1186/s13063-024-08157-y

**Published:** 2024-05-15

**Authors:** Danwei Zhang, Bei Zhao, Xue Sun, Kaimo Ding, Jingjing Sun, Sheng Tao

**Affiliations:** 1 Department of Psychology, Zhenjiang Mental Health Center, No. 199 Tuanshan Road, Zhenjiang, Jiangsu 212001 China; 2 Department of Psychiatry, Zhenjiang Mental Health Center, No. 199 Tuanshan Road, Zhenjiang, Jiangsu 212001 China

**Keywords:** High-definition transcranial direct current stimulation, Augmentation therapy, Major depressive disorder, Anxious distress specifier

## Abstract

**Background:**

Comorbid anxiety disorders and anxious distress are highly prevalent among individuals with major depressive disorder (MDD). The presence of the DSM-5 anxious distress specifier (ADS) has been associated with worse treatment outcomes and chronic disease course. Few studies have evaluated the therapeutic effects of High-definition transcranial direct current stimulation (HD-tDCS) on depressive and anxiety symptoms among MDD patients with ADS. The current randomized controlled trial aims to assess the efficacy of HD-tDCS as an augmentation therapy with antidepressants compared to sham-control in subjects of MDD with ADS.

**Methods:**

MDD patients with ADS will be recruited and randomly assigned to the active HD-tDCS or sham HD-tDCS group. In both groups, patients will receive the active or sham intervention in addition to their pre-existing antidepressant therapy, for 2 weeks with 5 sessions per week, each lasting 30 min. The primary outcome measures will be the change of depressive symptoms, clinical response, and the remission rate as measured with the 17-item Hamilton Depression Rating Scale (HDRS-17) before and after the intervention and at the 2nd and 6th week after the completed intervention. Secondary outcome measures include anxiety symptoms, cognitive symptoms, disability assessment, and adverse effects.

**Discussion:**

The HD-tDCS applied in this trial may have treatment effects on MDD with ADS and have minimal side effects.

**Trial registration:**

The trial protocol is registered with www.chictr.org.cn under protocol registration number ChiCTR2300071726. Registered 23 May 2023.

**Supplementary Information:**

The online version contains supplementary material available at 10.1186/s13063-024-08157-y.

## Introduction

### Major depressive disorder with anxious distress exerts a significant impact on the healthcare system

To emphasize the importance and impact of the concurrence of anxiety and depressive disorders, the Diagnostic and Statistical Manual of Mental Disorders-5th edition (DSM-5) has added the anxious distress specifier (ADS) [1]. The diagnostic criteria for ADS require the presence of at least two of the following symptoms: (1) feeling keyed up or tense, (2) restlessness, (3) difficulty concentrating due to worry, (4) feeling that something awful may happen, and (5) feeling that one may lose control of oneself. Data suggests that ADS criteria are met by 50–65% of individuals diagnosed with major depressive disorder (MDD), and the specifier is more effective than comorbid anxiety diagnoses in predicting significant clinical outcomes such as chronicity, remission, and disability [2–5]. This underscores its presumed clinical relevance. Greater severity of illness was observed 

in adults with ADS, as evidenced by a higher frequency of hospitalizations, increased rates of suicidal ideation, greater severity of depressive symptoms, more significant workplace impairment, reduced quality of life, and self-reported cognitive decline [6]. Therefore, there is an urgent need for a novel treatment option that can effectively and tolerably address the needs of these subjects.

### Advantages of a new format of tDCS: HD-tDCS

A main drawback of traditional transcranial direct current stimulation (tDCS) is the spatial crudity of its effect. The highest cortical current density may not occur directly under the target electrode [7], which probably alters the effects of tDCS and contributes to the inconsistent results of existing tDCS studies [8, 9]. HD-tDCS was developed as a solution to tackle this issue with high precision and accuracy. Similar to conventional tDCS, which utilizes an anode and a cathode placed on the scalp to establish a unidirectional current flow, HD-tDCS also involves the use of both anode and cathode electrodes, typically consisting of more than two smaller electrodes. In the latter, precise delivery of current to the intended target site is its primary advantage. Although various montages can be explored, the “4X1 ring” montage is by far the most commonly used. It utilizes a center anode electrode surrounded by four return cathode electrodes and confers HD-tDCS with the ability to achieve a single desired activity without inducing cortical stimulation due to opposite polarity [10, 11]. The density of the cortical field and spatial focality can be modulated by changing the diameter of the ring of electrodes [12]. In addition to enhancing the spatial focality of stimulation, the augmented effects of neuroplasticity may also manifest in longer-lasting after-effects, as demonstrated in a previous study on HD-tDCS [10]. At the same time, the tolerability was found to be comparable to that of conventional tDCS [13]. No HD-tDCS study of MDD with ADS has yet been performed. Therefore, our team conducted a pilot study to test the effects and tolerability of HD-tDCS on MDD patients with ADS [14].

### Results from our pilot study

A pilot study [14] was conducted by our team in 39 adults who have MDD with ADS received five consecutive daily sessions of 20-min active or sham HD-tDCS interventions weekly for 2 weeks. The results revealed that the HD-tDCS has significant efficacy in the treatment among MDD patients with anxious distress. The rate of adverse events(e.g., itching, tingling and headache) showed no significant differences between the two groups. Additionally, no severe adverse events or mania was reported. Nevertheless, it should be noted that the trial was of short duration, limited to hospitalized patients, and did not include an evaluation of cognitive or daily functioning. A double-blinded randomized controlled trial (RCT) with an adequate sample size and adequate study periods is warranted to confirm the efficacy of HD-tDCS as an augmentation therapy with antidepressants in MDD patients with ADS.

### Objectives

This study proposes a RCT with parallel group design, in which participants are randomly allocated to the experimental group or the sham group in a 1:1 ratio. This trial intends to investigate the superiority of HD-tDCS treatment over sham stimulation. It is hypothesized that MDD patients with ADS receiving ten consecutive sessions of HD-tDCS will report a positive effect on the impact of on depressive symptom and confirm the findings of our pilot investigation, compared to patients receiving sham stimulation.

### Ethics and dissemination

The study is being conducted in accordance with the Declaration of Helsinki and was approved by the local ethics committee of Zhenjiang Mental Health Center (ethics approval number: 2023Y01) on April 26, 2023; then, the ethics committee’s phone, email, and address are as follows: 0086-0511-88773868, zjwyllwyh@163.com, and No.199, Tuanshan Road, Runzhou District, Zhenjiang 212000, China. Investigators will obtain signed informed consent forms from participants prior to their enrollment in the study. Following the study, all patients will continue to receive ongoing medical care from the investigators. The findings of this research will be disseminated through scholarly articles and presentations at national and international conferences. Therefore, this study will make a valuable contribution to the current treatment options for patients with MDD and offer a non-pharmacological intervention approach that will be readily available in the near future.

### Trial design

The study described in this protocol is a RCT to evaluate the superiority of an active HD-tDCS program for MDD patients with ADS compared with the sham HD-tDCS program. MDD patients with ADS will undergo at least 2 weeks of maintenance pharmacotherapy prior to stimulation initiation and continue such treatment throughout the entire stimulation period. Fifty-eight MDD patients with ADS will be randomized (1:1) in the active HD-tDCS group or the sham HD-tDCS group.

## Methods

### Recruitment

**Fig. 1:**
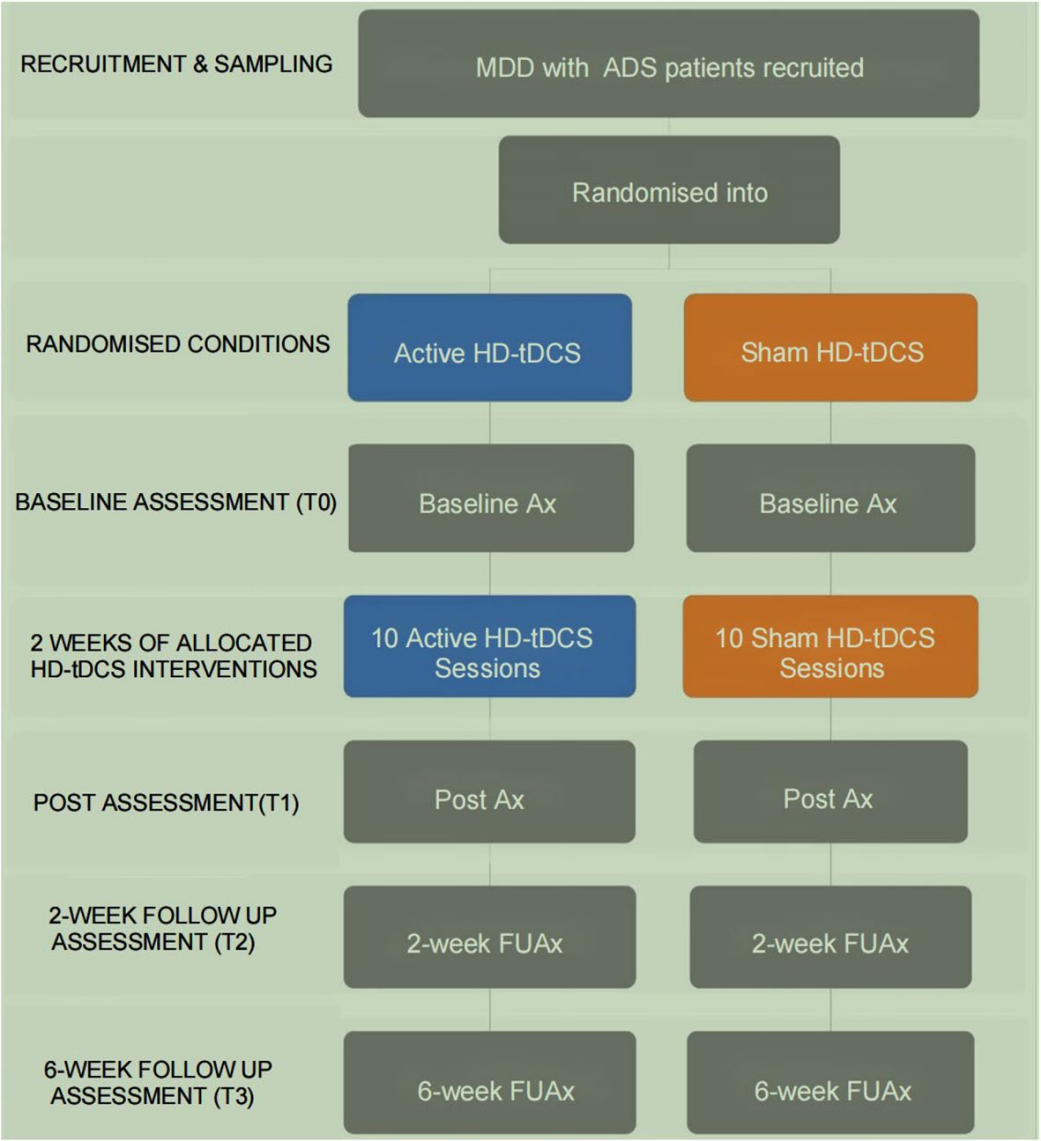
Flowchart of the current study

We will approach patients in the Zhenjiang Mental Health Center outpatient and inpatient units. Our inpatient and outpatient care units follow up at least 5000 patients with MDD each year. We will ensure the completion of the study schedule in accordance with the current protocol. Standard clinical care will continue to be provided to all participants upon completion of the trial at our healthcare facility. Recruitment, pre-participation screening, allocation, intervention, and data collection will be conducted between Jun 2023 and November 2025. Our plan is to conclude the study by the end of 2026 and make the results available for analysis. The current study protocol adheres to the SPIRIT 2013 recommendations (Standard Protocol Items: Recommendations for International Trials) [15]. Figure 1 shows the schedule of enrollment, interventions, and assessments.

### Eligibility criteria

The inclusion criteria are as follows:

1. Ability and willingness to provide written informed consent for trial participation and undergo all necessary procedures is required;
2. The age range is between 18 and 65 years old;
3. A diagnosis of MDD with ADS according to the Diagnostic and Statistical Manual of Mental Disorders, Fifth Edition (DSM-5), which is assessed by at least one professional psychiatrist; the presence of specifier was evaluated based on DSM-5 criteria, which mandates that MDD patients exhibit at least two out of five symptoms;
4. Han ethnicity;
5. Right-handedness;
6. Adequate auditory and visual acuity to perform requisite inspections;
7. Presence of depressive symptoms ranging from mild to severe, as measured and defined by HDRS-17 scores ≥ 8 and ≤ 52 [16];
8. Willing to remain on maintenance pharmacotherapy for at least 2 weeks before the stimulation initiation and during the total stimulation period.

The exclusion criteria are as follows:

1. The presence of severe systemic illnesses, including but not limited to severe hepatic, renal, endocrine, cardiovascular, respiratory, hematological, or oncological diseases that are deemed by the investigator as unsuitable for participation in the trial;
2. Any laboratory examination that shows clinically significant abnormalities and may have an impact on the health of participants, as determined by the investigator;
3. A history of any significant medical illness, such as neurological disorders (e.g., cerebral trauma, seizure disorder, etc.), that would render the participant unsafe for trial participation in the investigator’s opinion;
4. Known to have current psychosis as determined by the DSM-5 criteria or a history of a non-mood psychotic disorder;
5. Current substance abuse, including alcohol and/or illicit or prescription drugs;
6. Current or planned pregnancy and/or lactation during the trial;
7. A scalp with abnormal conditions, such as open wounds;
8. A score = 4 for item 3 (suicide) on the HDRS-17;
9. Having undergone or currently undergoing modified electroconvulsive therapy (MECT) or repetitive transcranial magnetic stimulation (rTMS) within the past month;
10. Concurrent participation in another clinical trial or participation within 1 month prior to randomization is not allowed.

### Randomization, concealment, and blinding

The eligibility participants will be randomly assigned to either active or sham HD-tDCS group with a 1:1 allocation. This study will use the Statistical Analysis Software (version 9.3, SAS Institute Inc., Cary, NC, USA) to generate the random allocation sequence. The randomization list will be enclosed in computer-generated opaque envelopes, each labeled with a sequence number on the outside. After the eligible patients have been screened, the researchers will proceed to open the envelopes. The unblinded researchers will create the randomization sequence and allocate participants to treatments. The trained operators responsible for administering HD-tDCS will not be blinded to allocation and, as a result, will be excluded from assessments and data processing. The participants, statisticians, and evaluators will be kept blinded to the treatment allocation. The unblinding process should only be initiated in the event of an emergency, such as the occurrence of any serious adverse events.

### Sample size calculation

The sample size will be determined using G*Power 3.1, based on the findings of a a tDCS meta-analysis of adult patients with MDD [17]. In this study, active tDCS was found to be significantly more effective than sham tDCS for the reduction of depression severity (Hedges’ *g* = 0.743). For a power of 80% and an alpha level of 5%, a total sample size of 48 patients (24 in each group) is required to detect significant differences between the groups. To account for a 20% drop-out rate, a total of 58 patients (29 in each arm) will be recruited.

## Interventions

### High–definition transcranial direct current stimulation (HD-tDCS) group

Trained nurses will administer the intervention and provide instructions for participants to receive either active or sham HD-tDCS treatment (Soterix medical, Inc., Woodbridge, NJ, USA) under the same instructions during all sessions for 2 weeks. All participants will be treated using the same device model. The HD-tDCS device is capable of being portable and wirelessly controlled through computer software developed by the manufacturer. The montages will be arranged in a “4 × 1 ring set-up,” which is the most commonly utilized HD-tDCS setting. The center anode electrode is surrounded by four return cathode electrodes. The anode will be positioned over the left DLPFC, which is located at F3 according to the 10/20 electroencephalogram system. The four cathodal electrodes will be placed at FC1, AF3, F7, and FC5 (Additional file 1). The designated electrode stimulation areas on the scalp will be coated with conductive electrode gel. The electrodes will be secured in place by using caps that are appropriate for the head size of each participant. Impedance checks will be performed prior to each treatment session. A 2-mA stimulation will be administered for a duration of 20 min, with the current gradually ramping up and down over a period of 30 s. The patients will be instructed to maintain a relaxed and immobile state throughout the intervention. The administrator will closely monitor the impedance levels throughout each session and document any adverse reactions reported by the participants. The participants will be granted a 5-min rest period following the intervention and will be actively queried regarding any discomfort they may experience. Each session will have a duration of approximately 30 min, and there will be a total of 10 sessions (consisting of two consecutive weeks of treatment, with 5 days per week).

### Sham–control HD-tDCS group

The procedure for sham stimulation will be identical, with the exception that the electrical current will gradually decrease to zero after 30 s, thereby producing an equivalent initial sensation of HD-tDCS. The stimulator will be programmed to automatically control the current, eliminating the need for operator intervention. The computer will be positioned posterior to the subjects’ craniums, thereby obstructing their visual access to the readout.

#### Criteria for discontinuing or modifying allocated interventions

There will be no special criteria for discontinuing or modifying allocated interventions.

#### Relevant concomitant care permitted or prohibited during the trial

Implementing HD-tDCS treatment or sham-controlled antidepressant augmentation will not require alteration to usual care pathways (including use of any medication), and these will continue for both trial arms.

#### Provisions for post-trial care

The patients will receive notification of their allocated arm (treatment or sham) at the conclusion of the study. Furthermore, participants will continue to receive standard care as determined by the treating psychiatrist or care team. It typically entails a combination of behavioral therapies, psychosocial interventions, and psychotropic medications.

## Outcomes

### Diagnostic interview

The diagnoses will be confirmed through a semi-structured psychiatric diagnostic assessment, conducted by an experienced psychiatrist using the validated Chinese-bilingual version of the Structured Clinical Interview for DSM Mental Disorders in accordance with DSM-5.

### Demographic data

The participants’ fundamental demographic information, encompassing age, gender, educational attainment, birthplace, marital status, offspring count, financial standing, family history of affective disorders, and household income will be obtained upon enrollment into the study. Details of the subjects’ psychiatric history, including the age at onset of their initial depressive episode, number of relapse episodes, and current medication and dosage, will be documented during the baseline assessment. The medical history and treatment of patients will be evaluated through direct inquiry and confirmed with their electronic health records in the hospital authority.

### Primary outcome

The primary outcome will be the change of depressive symptoms. The clinical response rate and remission rate, as assessed by the HDRS-17 (17-item Hamilton Depression Rating Scale) at 6 weeks post-intervention were measured. The Chinese version of HDRS-17 has been validated for its psychometric properties and is commonly used to evaluate depressive severity in Chinese patients with depression symptoms [18]. The clinical response is defined as a ≥ 50% reduction from the baseline [19], and a score of 7 or less will be used as an indicator of remission [20].

### Secondary outcomes

#### Anxiety symptoms

Anxiety symptoms will be measured by the DSM-5 Anxious Distress Specifier Interview (DADSI), which is more brief than the HAMA (Hamilton Anxiety Scale) and may prove to be a more practical tool in clinical settings [21]. The DADSI probes evaluate the five symptoms of the anxious distress specifier and inquire about symptom presence and severity over the past week, which also determine whether the symptom is present for a majority of the depressive episode. Item severity for the past week is rated from 0 to 4 (0 = not at all true, 1 = rarely true, 2 = sometimes true, 3 = often true, 4 = almost always true) [22]. The DADSI is a reliable and valid measure for assessing the presence of the DSM-5 anxious distress specifier in individuals with MDD as well as evaluating the severity of its associated features [22]. Both the DADSI and HAMA were valid measures of anxiety severity in depressed patients; however, the HAMA exhibited a higher degree of confounding with depression measures compared to the DADSI [21]. Earlier studies [4, 23] have shown that the ADS exhibits satisfactory internal consistency (Cronbach’s = 0.71) and predictive validity for subsequent course and treatment response in depressed patients [24].

#### Cognition

For the assessment of cognitive status in MDD patients with ADS, a battery of neurocognitive tools such as the Repeatable Battery for the Assessment of Neuropsychological Status (RBANS) [25] is utilized, encompassing various domains including attention, immediate and delayed memory, language, and visuospatial/constructional abilities. The Chinese version of RBANS has been certified as a reliable tool for assessing cognitive function with reasonable reliability and validity. Higher scores indicate better neurocognitive status.

#### Disability assessment/daily functioning

Disability was evaluated using the WHO Disability Assessment Schedule (WHODAS 2.0), a tool that has demonstrated good reliability and validity in assessing adults from diverse cultural backgrounds [26]. In China, disability caused by mental disorders is clearly defined based on the duration of the disorder and the individual’s score on WHODAS. The WHODAS 2.0 full version comprises of 36 questions that evaluate six domains, namely cognition (communication and thinking activities), mobility (standing, moving around inside the home, getting out of the home, and walking a long distance), self-care (hygiene, dressing, eating, and staying alone), getting along with others (interactions with other people), life activities (domestic responsibilities, leisure, work, and school), and participation (joining in community activities and participating in society).

#### Adverse effects and risk indicators

The Young Mania Rating Scale (YMRS) [27] will be utilized to detect treatment-induced manic or hypomanic episodes, an 11-item observer-administered scale of mania intensity. YMRS ranges from 0 to 60, with higher scores indicating greater severity. The minimum score required to define a new-onset mania or hypomania is 8 points. A checklist of potential adverse effects associated with the administration of HD-tDCS will be compiled based on available literature [28, 29]. The checklist (Additional file 2) will be utilized to monitor the tolerability and adverse events in each session throughout the intervention. The assessment of risk indicators, such as suicidal ideation or severe self-neglect, will be conducted directly to determine the need for immediate changes in treatment based on identified risks and needs.

### Planned visit schedule

The visit schedule for all assessments is presented in Table 1.

**Table 1:** Data collection methods and clinical assessment time points

Timepoint	Screening	T0	T1	T2	T3
**Enrolment:**					
Signed informed consent		X			
Diagnosis	X				
Randomization		X			
**Interventions:**					
Sham intervention group		X	X		
Active intervention group		X	X		
**Assessments:**					
*Primary outcome*					
HDRS-17	X	X	X	X	X
*Secondary outcomes*					
DADSI		X	X	X	X
RBANS		X	X	X	X
WHODAS 2.0		X	X	X	X
*Adverse effects*					
YMRS	X		X	X	X
Adverse checklist			X		
Pregnancy test	X				
Patients’ compliance			X	X	X
Blinding assessment					X

X represents data collection timepoint

*HDRS-17* 17-item Hamilton Depression Rating Scale, *DADSI* the DSM-5 Anxious Distress Specifier Interview, *RBANS* the Repeatable Battery for the Assessment of Neuropsychological Status, *WHODAS 2.0* the WHO Disability Assessment Schedule 2.0; *YMRS*, the Young Mania Rating Scale

### Statistical analysis

Continuous variables will be summarized using the mean and standard deviation measures. Categorical variables will be presented in terms of frequency and percentage. Between-group comparisons will be assessed using the Mann–Whitney *U* test for continuous variables and either the chi-square test or Fisher exact test for categorical variables. The primary outcome will be analyzed based on the intention-to-treat principle, with worst case imputation applied. For repeated continuous outcomes, a mixed linear model will be employed, which includes the interaction between group (active versus sham) and time (baseline, post-assessment, 2-week and 6-week follow-up assessment.

The hypotheses will be tested using unpaired, two-tailed tests at a significance level of 0.05. The software SAS, v.9.4 (SAS Institute Inc.), was utilized.

## Oversight and monitoring

### Composition of the coordinating center and trial steering committee

A research management group, consisting of the trial investigators, will be established to oversee the project’s governance and day-to-day operations. The group will meet on a monthly basis to assess progress and ensure that the study is being conducted in accordance with the requirements of research ethics approval. While the importance of a trial steering committee in upholding study conduct quality is recognized, financial limitations have prevented the establishment of such a committee. Nevertheless, this research will be carried out under the auspices of Zhenjiang Mental Health Center, where stringent research governance structures are in place to ensure the quality of study conduct.

### Composition of the data monitoring committee, its role, and reporting structure

The research management group will take on the responsibility of data monitoring. Although we acknowledge that an independent committee would be optimal for this role, financial constraints have made it unfeasible to appoint one. However, as mentioned earlier, stringent research governance procedures are in place at Zhenjiang Mental Health Center to ensure the quality of study conduct.

### Adverse event reporting and harms

tDCS is considered to be a safe technique, but some small risks are recognized. A checklist of potential adverse effects associated with t-DCS administration will be generated with reference to the available literature [28, 29]. All potential risks will be fully disclosed to the participants, along with instructions on how to handle any side effects they may experience. Furthermore, participants will receive a debriefing at the conclusion of each session and will be instructed to contact the principal investigator in the event of any adverse effects resulting from their involvement in the study. In the event of a serious adverse reaction during treatment sessions, local staff (clinic, hospital, institutional) will be promptly notified and emergency protocols will be implemented. We do not expect any adverse events, whether serious or otherwise. However, in the event that one does occur, it will be documented and reported to the local ethics committee of Zhenjiang Mental Health Center.

### Plans for communicating important protocol amendments to relevant parties (e.g., trial participants, ethical committees)

The local ethics committee of Zhenjiang Mental Health Center will be consulted for approval of significant protocol modifications, such as changes to eligibility criteria, outcomes, and analyses. Upon receipt of approval, the modifications will be formally communicated in written form to relevant stakeholders (such as investigators and trial participants), and the trial record on chictr.org.cn will be appropriately updated.

### Auditing

The principal investigator (PI) ST shall assume responsibility for the ongoing management of the study. The sponsor shall oversee and perform audits on a subset of studies within its clinical research portfolio. The monitoring and auditing activities will be carried out in accordance with the sponsor’s established procedures for monitoring and audit.

## Discussion

In the DSM-5, an ADS has been included for the diagnosis of MDD [30], which is common among MDD patients without comorbid anxiety disorders and correlated with some of their sociodemographic and clinical characteristics [4]. It captures the core symptoms of anxious emotions and cognitions, providing a valuable framework for treatment planning and symptom assessment [4]. The ADS predicts poorer treatment outcomes as shown by higher depression severity, lower remission rates, and greater frequency of antidepressant side effects in patients with MDD on adequate antidepressant treatment; in contrast, the presence of comorbid anxiety disorders did not predict these treatment outcomes [4]. The assessment of anxiety distress according to DSM-5 was found to be significant not only during the acute phase but also in the continuation/maintenance phase of MDD [31]. This simple 5-item specifier holds great potential for clinical utility in the planning and monitoring of treatment for depressed patients; therefore, new and effective interventions beyond pharmacotherapy and psychotherapy are imperative for this subpopulation with heightened illness-associated burden and risks [6].

This randomized controlled trial aims to evaluate the effectiveness of HD-tDCS as augmentation therapy with antidepressants in ameliorating depressive symptoms in MDD patients with ADS. To this aim, the trial compares the effect of HD-tDCS and sham-control HD-tDCS on improving depressive symptoms in addition to the pre-existing antidepressant regimen. The operationalization of depressive symptoms encompasses three levels, specifically the change in depressive symptoms, clinical response, and remission rate as assessed by HDRS-17. The secondary objectives of this study are to evaluate the impact of the intervention on anxiety symptoms, cognitive function, disability resulting from mental disorders, and adverse effects upon completion of the intervention. We also plan to detect any sustained effect by checking the changes at the 2nd week and 6th week after the finished intervention.

The findings of this trial will prove valuable in the management of MDD patients with ADS, as a new safe treatment option is required to alleviate depressive symptoms for those exhibiting inadequate responses to conventional antidepressants. HD-tDCS is anticipated to be efficacious in ameliorating cognitive function and daily functioning, which are frequently compromised in individuals with MDD.

We acknowledge some limitations of the trial. The study was conducted at a single center, and the generalizability of its findings to other regions or ethnic groups may be limited. In our future studies on HD-tDCS, we shall strive to provide a novel medical foundation for treating MDD patients with ADS using HD-tDCS. The duration of the intervention is brief, and currently, the optimal frequency of presentation to patients remains unknown for achieving maximum efficacy. Although the dosage and timing of antidepressants will remain unchanged for at least 2 weeks prior to the study, it is possible that a change in medication may have an impact, potentially leading to an overestimation of the effect by HD-tDCS. Meanwhile, the diversities of clinical profiles may serve as potential confounding factors, including the types of antidepressants utilized, suboptimal treatment response and resistance levels, or duration of depressive symptoms. Therefore, several key questions remain unanswered and warrant further investigation. These include determining whether patient characteristics or other variables have an impact on outcomes and to what extent. Subgroup analysis will be conducted to mitigate the impact of confounding factors and enhance the trial’s validity.

In conclusion, this trial is a pragmatic randomized double-blinded sham-controlled study that seeks to compare the impact of HD-tDCS versus sham HD-tDCS in treating MDD patients with ADS. To our knowledge, this study is the first HD-tDCS protocol of in this patient population and the first to employ detailed measures of anxiety symptoms combined with assessment of cognition and daily functioning, which will also be used in future analyses. The anticipated outcomes of this investigation hold significance, as it will offer a non-pharmacological intervention for this condition with minimal adverse effects.

## Trial status

Protocol version: 5 August 2023, version no. 1

Date of recruitment: 26 April, 2023

End of recruitment: estimated to be December 2025

### Supplementary Information

Additional file 1. HD-tDCS setting of electrode annotations.

Additional file 2. SPIRIT checklist.

## Data Availability

During the current phase of data collection, researchers will have limited access to the data. After the study is completed, access to the data will be restricted to only the principal investigator (ST) and study coordinator (DZ). Due to privacy concerns, it is not feasible to make raw data openly accessible to the public. Upon request to the corresponding author, access will be granted to the data, statistical parameters, and statistical code.

## Abbreviations

DSM-5: Diagnostic and Statistical Manual of Mental Disorders-5th edition
ADS: Anxious distress specifier
MDD: Major depressive disorder
tDCS: Transcranial direct current stimulation
HD-tDCS: High-definition transcranial direct current stimulation
RCT: Randomized controlled trial
SPIRIT: Standard Protocol ItemsRecommendations for International Trials
HDRS-17: 17-Item Hamilton Depression Rating Scale
DADSI: The DSM-5 Anxious Distress Specifier Interview
RBANS: The Repeatable Battery for the Assessment of Neuropsychological Status
WHODAS 2.0: The WHO Disability Assessment Schedule 2.0
YMRS: The Young Mania Rating Scale
