# Supplementary figures and images for: High-definition transcranial direct current stimulation (HD-tDCS) in major depressive disorder with anxious distress—a study protocol for a double-blinded randomized sham-controlled trial
Source: Trials. 2024 May 15;25:320. doi: 10.1186/s13063-024-08157-y (PMC11094846; doi:10.1186/s13063-024-08157-y)

Additional file 1. HD-tDCS setting of electrode annotations

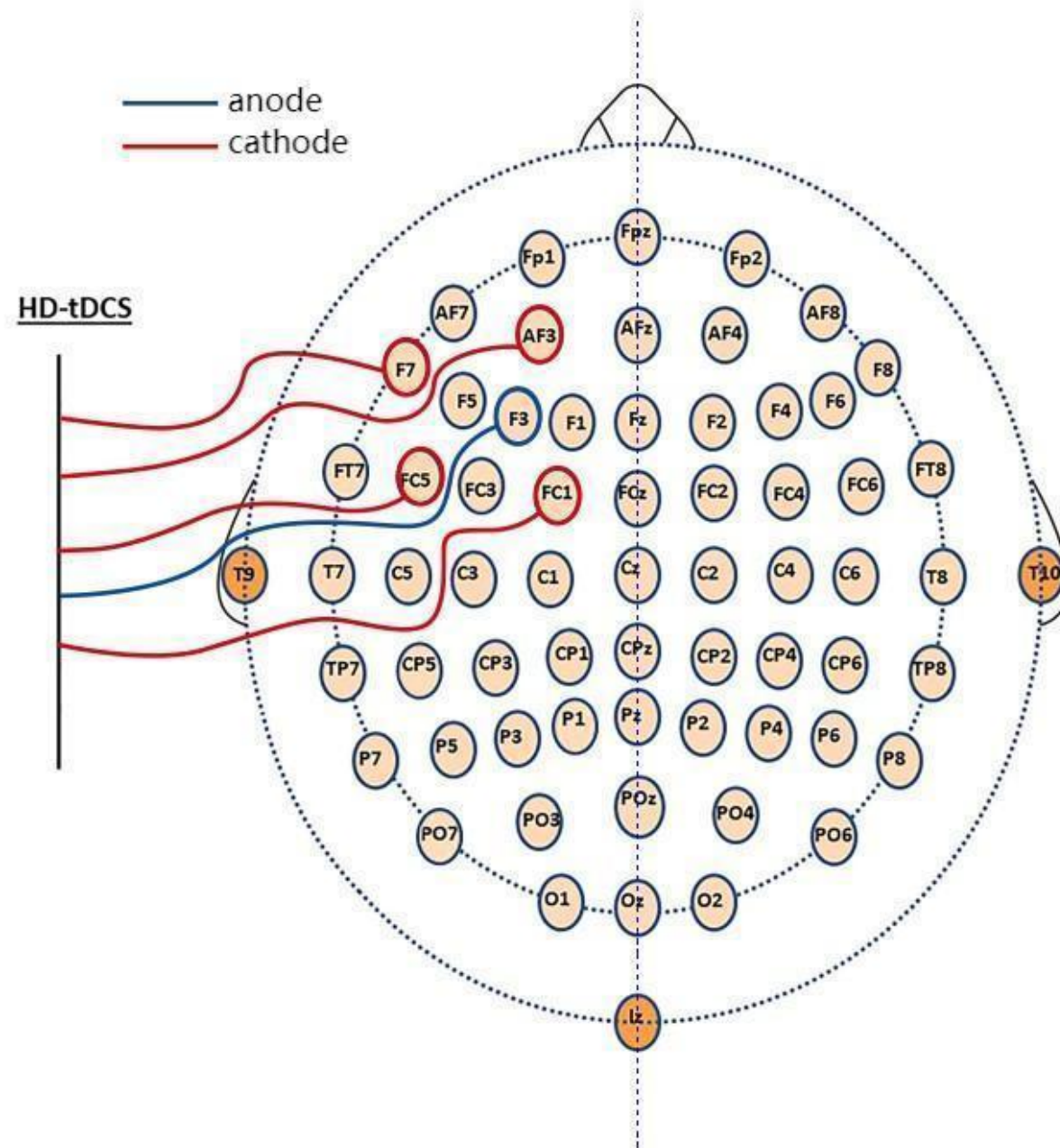

Supplement: Supplementary file 1 — Additional file 1. HD-tDCS setting of electrode annotations. [file 13063_2024_8157_MOESM1_ESM.pdf]
